# Improving hand hygiene compliance in the emergency department: getting to the point

**DOI:** 10.1186/1471-2334-13-367

**Published:** 2013-08-07

**Authors:** Simone Scheithauer, Vanessa Kamerseder, Peter Petersen, Jörg Christian Brokmann, Luis-Alberto Lopez-Gonzalez, Carsten Mach, Roland Schulze-Röbbecke, Sebastian W Lemmen

**Affiliations:** 1 Department of Infection Control and Infectious Diseases, RWTH Aachen University Hospital, Aachen, Germany; 2 Department of Emergency Medicine, RWTH Aachen University Hospital, Aachen, Germany; 3 Department of Medical Microbiology and Hygiene, University Hospital, Düsseldorf, Germany

## Abstract

**Background:**

The emergency department (ED) represents an environment with a high density of invasive, and thus, infection-prone procedures. The two primary goals of this study were (1) to define the number of hand-rubs needed for an individual patient care at the ED and (2) to optimize hand hygiene (HH) compliance without increasing workload.

**Methods:**

Prospective tri-phase (6-week observation phases interrupted by two 6-week interventions) before after study to determine opportunities for and compliance with HH (WHO definition). Standard operating procedures (SOPs) were optimized for invasive procedures during two predefined intervention periods (phases I and II) to improve workflow practices and thus compliance with HH.

**Results:**

378 patient cases were evaluated with 5674 opportunities for hand rubs (HR) and 1664 HR performed. Compliance significantly increased from 21% (545/2603) to 29% (467/1607), and finally 45% (652/1464; all p<0.001) in phases 1, 2, and 3, respectively. The number of HR needed for one patient care significantly decreased from 22 to 13 for the non-surgical and from 13 to 7 for the surgical patients (both p<0.001) due to improved workflow practices after implementing SOPs. In parallel, the number of HR performed increased from 3 to 5 for non-surgical (p<0.001) and from 2 to 3 for surgical patients (p=0.317). Avoidable opportunities as well as glove usage instead of HR significantly decreased by 70% and 73%, respectively.

**Conclusions:**

Our study provides the first detailed data on HH in an ED setting. Importantly, HH compliance improved significantly without increasing workload.

## Introduction

### Background

Hand hygiene (HH) is the cornerstone in infection prevention and infection control [1]. Currently, studies on HH in an emergency department (ED) setting are limited [2-4]. Notably, there are no data on HH opportunities, hand rubs (HR), or compliance according to the WHO recommendations for individual patient care in an ED setting.

### Importance

Healthcare-associated infections (HCAIs) have a great impact on morbidity, length of hospital stay, and treatment costs [5]. The use of invasive devices is one of the most important risk factors for acquiring HCAIs [5]. The ED represents an environment with a high density of invasive, and thus, infection-prone procedures [6].

HH is considered to be the single most effective tool to prevent HCAIs [1,7]. WHO defined “five moments” for HH and highlighted the need for new strategies to improve everyday HH practices on the basis of the current low compliance [8]. Factors negatively influencing compliance with HH are well studied and many are prevalent in an ED setting [1,8]. For example, the work conditions of an ED make it particularly prone to high workload and patient turnover, crowding of patients and health care workers (HCW), and HCW variety [2].

### Goal of this investigation

We conducted a prospective tri-phase (6-week observation phases interrupted by two 6-week interventions) before after study to determine HH quality and to identify critical factors underlying low compliance. Our interventions were aimed at increasing the number of HR as well as reducing the number of HR needed by improving workflow practices.

We hypothesized that an intervention focused on optimizing workflow would simplify work by not increasing - and even reducing - workload.

## Methods

### Study design and setting

We performed a prospective, tri-phase before after study in the ED of the RWTH Aachen University Hospital between February 2011 and September 2011. Healthcare-workers observed were classified into the four groups: physicians, nurses, medical students, trainees (nurses, paramedics) and analysis was stratified accordingly.

**Figure 1:**
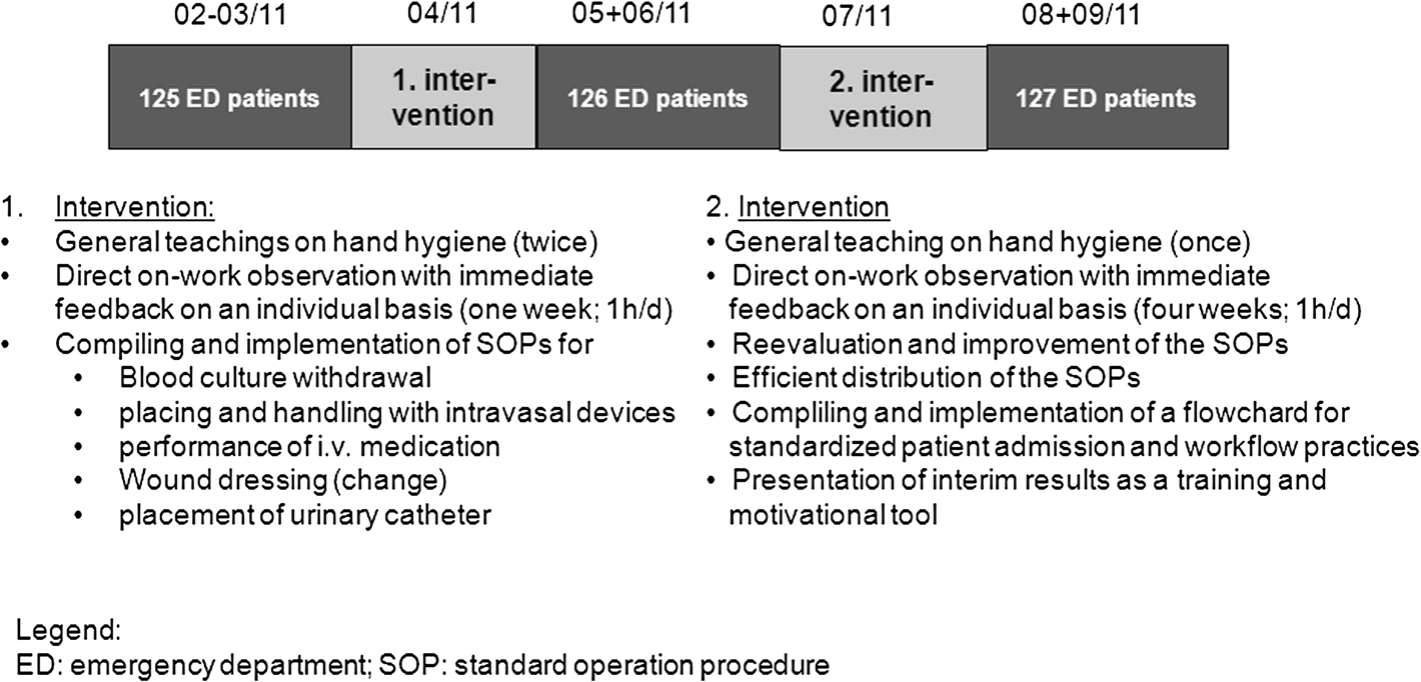
Study design, timeline and interventions.

The study consisted of three 6-week observation phases interrupted by two 6-week interventions (for timelime see Figure 1).

Documentation was performed anonymously and no patient-directed interventions occurred. The investigation was part of the infection control quality management of the institution; therefore no informed consent was taken. All health care workers gave their consent orally.

### Selection of participants

Patients were continuously enrolled in the study with five patients a day, respectively. A similar subgroup enrollment as well as a large health-care worker variability were taken into consideration. Patients were categorized in four groups: medical-thoracic, medical-abdominal, neurological, and surgical patients. 50, 51, and 50 surgical patients and 75, 75, and 77 non-surgical patients were enrolled during each phase (1, 2, and 3) with at least 25 for each non-surgical sub-group, respectively. A total of Only cardiopulmonary resuscitations were excluded.

Every patient was included only once; after each inclusion the health-care worker primarily observed was changed to cover the widest range of patients and health-care workers.

The local ethics committee did not request an ethics approval since the investigation was part of the quality and patient safety effort of the infection control team and did not include interventions on patient level and documentation was performed anonymously.

### Interventions

Interventions were targeted at problems that had been identified during the preceding observation phase and were implemented by the infection control staff (two infection control nurses, two infection control physicians and one medical student) in collaboration with the ED team (nurses and physicians). Interventions mainly consisted of individual teachings on work performance with direct feedbacks (1h/ day during the intervention phases), development and implementation of standard operation procedures (SOPs), and the compilation of flowcharts for patient admission. The on work teachings including feedback were performed for each staff member available during the intervention. SOPs aimed at omitting avoidable HR opportunities mainly due to recontaminations within one procedure and as a consequence, optimizing workflow. SOPs addressed placement and handling of intravenous and urinary catheters, blood withdrawal, as well as management of wound dressings, and were widely distributed, e.g. as pocket cards (Additional file 1). The flowchart aimed at workflow optimization and standardization in order to improve efficacy and reduce opportunities for HR (for interventions see Figure 1).

### Methods and measurements

HH indications 1–5 were classified according to the WHO guideline [1]. According to these recommendations, HR are needed (=opportunity) before touching a patient (1), before aseptic tasks (2), after contact with body fluids (3), after contact with a patient (4), and after contact with the patient’s closest surrounding (5). HR were recorded independently from the use of sterile or non-sterile gloves [1]. Glove usage instead of hand-rubbing was documented as well as HR performed without indication. Additionally, opportunities derived from avoidable recontaminations were classified as avoidable opportunities, Moreover, if an opportunity could be avoided by a better organized workflow, it is classified as avoidable. For example: (1) hand disinfection (avoidable)– preparing of materials needed for blood puncture – hand disinfection (often missed) - blood puncture; (2) hand disinfection – wound dressing - preparing missing materials - hand disinfection (avoidable) – wound dressing. When two HH indications were observed simultaneously, the procedure with the theoretically higher impact was recorded, e.g. before aseptic tasks (2), not before patient contact (1). If an opportunity for HH was given for two reasons (=indications) simultaneously, it was documented as one opportunity, but subsequently analyzed for both indications.

Documentation was performed using a modified version of the WHO observation record. Direct observation was performed anonymously by only one highly trained observer. HH observations were regularly conducted by the infection control team. The study started after a 6-weekpilot phase (1) to ensure familiarization with the situation of being observed, (2) to adapt WHO definitions to the specific demands of the ED setting, and (3) to minimize the effects because of observation.

Rooms had been equipped with at least one hand disinfectant dispenser long before the study started; no changes were made during the study period. Moreover, trainings on infection control had previously been performed on a regular and voluntary basis following good clinical practice.

### Outcomes

The main outcome parameter was HH compliance that is a widely accepted quality parameter [1]. Compliance rate (%) was calculated as the number of HR divided by the number of HR opportunities.

Secondary outcome parameters were (1a) the number of HR needed for an individual patient care and (1b) the number of avoidable opportunities as surrogate parameters for the workflow as well as (2a) the use of gloves instead of an indicated HR and (2b) HR without indications as additional quality parameters.

### Analysis

Statistical analyses were performed using SigmaStat 3.1.1 (Systat). The Fisher’s exact test and unpaired t-test were applied when appropriate, otherwise the Chi squared test with Yates correction and Wilcoxon Rank Sum test were performed.

Assuming a baseline compliance of 20% (calculated from the pilot phase before phase I) and calculating the potential for increase with 50% after each intervention (assumed goal after the first intervention: 30% and after the second one: 45%) with a given a given alfa-error level of 5% and a beta.error level of 20% (power: 80%), resulted in the need of 256 opportunities in each subgroup during the first two phases and 150 opportunities during phase 3, respectively. Before starting an inter observer agreement of at least 96% was achieved between each of the two infection control nurses and the V.K. performing the observation in the E.D. (number of observed agreements: at least 208 (96% of the observations); Kappa= 0.949; SE = 0.018; 95% CI: 0.915 to 0.984).

## Results

**Figure 2:**
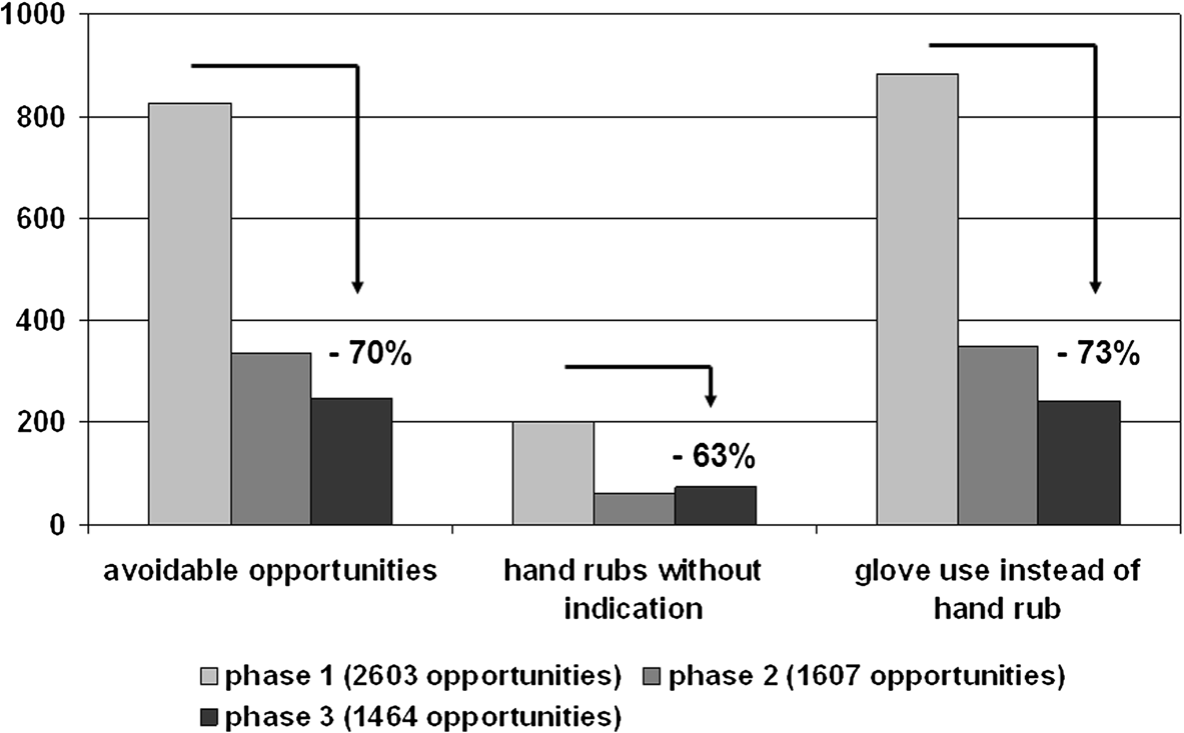
Decrease of common mistakes during the study.

A total of 5674 opportunities for HH and 1664 HR were recorded during the tri-phase study period. The number of opportunities steadily declined from phase 1 to phase 3 indicating a work-flow optimization. In contrary, the number of HR increased from phase1 to phase 3. Thus, resulting in a significant increase (p<0.001) in compliance from 21% to 45% over the three phases of the study (Figure 1). 125, 126, and 127 individual patient care episodes were included in the phases I, II, and III. In detail, 2603, 1607, and 1464 opportunities occurred during phase I, II, and III, and 546, 467, and 652 hand rubs were performed across the periods, respectively. Profession-specific analysis revealed an increase in all groups, but trainees (61 hand-rubs / 205 opportunities; compliance: 30% in phase 1; 33% in phase 3). Nurses started with 18% and reached 45% (overall hand-rubs/opportunities: 695/2448); medical students came from 20% and ended at 51% (overall hand-rubs/opportunities: 308/1141); physicians‘ compliance increased from 26% to 43% (overall hand-rubs/opportunities: 600/1889). Compliance revealed indication-specific differences with a range from 5% (indication 2) to 38% (indication 4) at baseline. The increase of compliance occurred for all indications. However, the greatest improvement was seem with indication 2 (660% of baseline), the lowest with indication 4 (150% of baseline), respectively. Subgroup analysis revealed no significant differences with the neurological and the medical-abdominal groups starting with 16% compliance and finally reaching 40% and 49%, respectively. The medical-thoracic and the surgical group started with 20% compliance both and ended at 44% and 42%, respectively. Hand rubs and opportunities split up between the patient groups as follows: 347/1378 in neurological patients; 379/1307 in medico-thoracic patients, 309/1079 in medico-abdominal patients, and 411/1442 in surgical patients, respectively. Analysis of the HR opportunities during phase 1 revealed a recurrent number of avoidable opportunities and “systematic mistakes” in workflow practices including re-contaminations after HR and/or not performing the HR *immediately* before an aseptic task. Optimizing the workflow by implementing SOPs resulted in a reduced number of avoidable opportunities and lower glove usage instead of HR (Figure 2).

For individual patient care, the number of HR needed significantly decreased from 22 to 13 for non-surgical and from 13 to 7 for surgical patients (both p<0.001) due to improved workflow practices resulting from the implemented SOPs.

**Figure 3:**
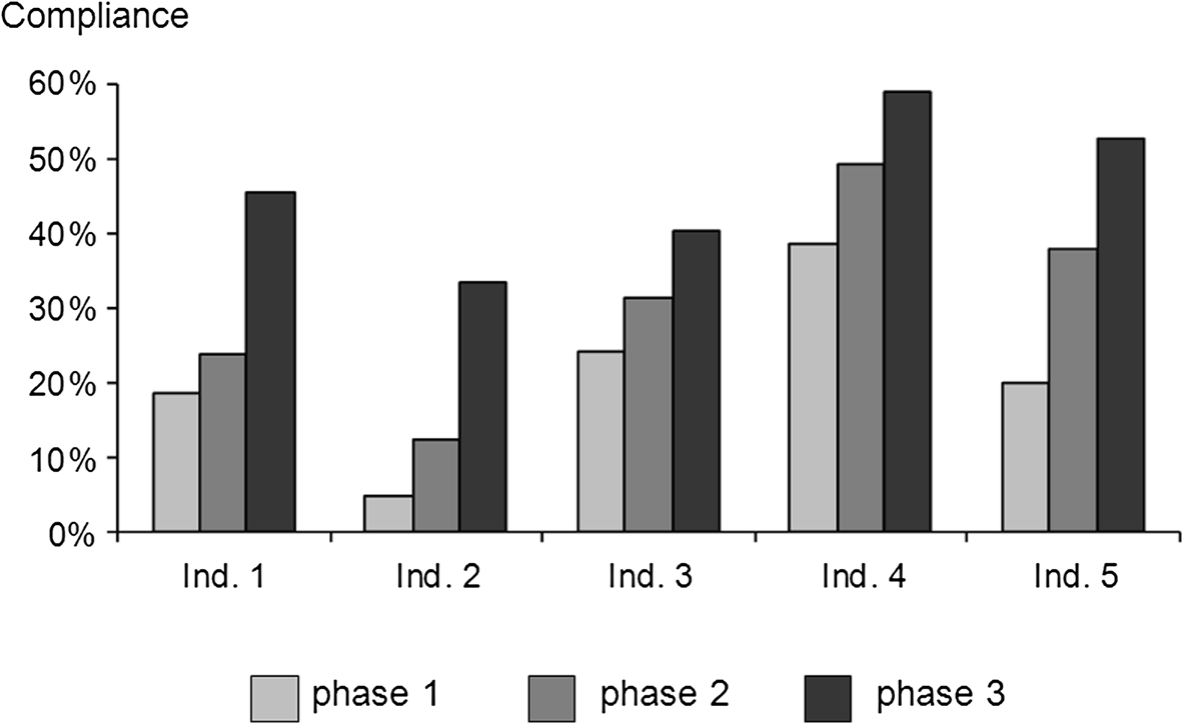
Compliance: indication-specific analysis (according to the WHO concept).

Compliance before patient contact and aseptic tasks (indications 1–2) was lower compared to that after patient contact (indications 3–5). However, the greatest increase was observed for indications 1 and 2 (Figure 3).

Of note was that no differences were documented for the different healthcare professions (physicians, nurses, medical students, trainee nurses) and for the different patient groups.

## Discussion

Our study offers the first detailed data on ED-related and indication-specific HH. The key finding of our study is that only a moderate increase in the number of HR performed in combination with a dominating decrease in the number of HR required significantly improved compliance without increasing workload. In other words: Reducing the number of hand rub indications increases compliance efficiently. Thus, introducing SOPs and training staff in these relatively easy to implement measures offers a feasible approach to improving overall quality of patient care in an ED – an approach that could also be translated into other hospital settings.

Our baseline HH compliance rate of 21% was considerably low compared with an average of 40% in the general healthcare field [1,8]. However, published compliance rates vary within a broad range of 5-98% and suggest lower rates in situations with a high activity level and/or involvement of physicians [1,8]. Notably, both of these factors are particularly prevalent in an ED. Currently, data on HH in an ED setting are very limited and results vary greatly. Early data from Meengs et al. (1994) revealed a handwashing compliance of 32% in the ED [2]. Interestingly, Di Martini et al. reported a baseline compliance of 14%, whereas Venkatesh et al. observed a 89% compliance rate [3,4]. However, both studies included handwashing as well an hand-rubbing as an HH activity, and therefore, a meaningful comparison is limited [3,4].

Nevertheless, a baseline compliance of 21% is unacceptable and raises the inevitable question as to why highly educated and trained healthcare workers perform HR so rarely. One major reason seems to be the limited amount of available time or the perception of limited time for preventive measures [1,7,8]. Thus, demanding an increase in the number of HR would further increase the workload. 11It is therefore of note that our recent study in a hemodialysis setting showed that by implementing SOPS, thereby improving workflow practices, compliance could be significantly increased by more than 100% [9].

For the ED setting, we found a significant reduction in the number of HR needed for individual patient care after implementing the SOPs and introducing the flowchart. As a result, compliance significantly increased by more than 100%. The higher compliance in phase 2 was a result of fewer HR needed, indicating that health care workers firstly implemented the time-saving part of the optimization program. Whereas, the even higher compliance in phase 3 was the result of both fewer HRs needed and more HR performed. Thus, standardization of workflow may be an appropriate way to improve the overall quality of HH and thus increase patient safety.

As shown for all [1,8] but one [10] healthcare setting, compliance before contacting the patient was lower than afterwards. HH before patient contact and before aseptic tasks (indications 1 and 2) plays a major role in controlling HCAIs and avoiding cross-transmission of (multi-resistant) bacteria, thus compliance with these two indications is a cornerstone in infection control [1,7]. In other words, the higher the necessity for HH, the lower the current adherence to recommendations. It is therefore of note that the highest increase in compliance observed in this study was for indications 1 and 2 with an up to 5.6-fold increase.

As been previously demonstrated glove usage was inversely correlated with adequate HH [1,11]. This bad practice decreased significantly albeit slowly, thereby indicating that individual training focused on this specific issue eventually worked; however, a high proportion of glove usage instead of HR still persisted even after phase 3. It is therefore surprising that despite these clear guidelines, it is hard to convince healthcare workers that gloves do not replace HR [11]. Disinfecting gloves might provide a solution in hospital settings with a high frequency of HR opportunities when caring for an individual patient like in an ED setting. However, there are currently no guidelines on recommending disinfecting gloves after a single use.

### Limitations

Since the study as performed at one ED (single center design) the question of transferability of the results remains unanswered. Moreover, the primary endpoint focused on hand hygiene and represented a quality parameter, not a clinical endpoint like device-associated infection rates which would improve the impact. In addition, possible overestimation of compliance due to the direct observation method – however currently regarded as the gold-standard (Hawthorne effect) has to be taken into consideration. And finally, the question of sustainability remains open. Despite we cannot provide a detailed cost analysis, increasing efficiency as a mechanism for improvement has been demonstrated.

## Conclusion

In conclusion, our study is the first to present detailed data on HH opportunities, HR, and compliance for individual patient care in an ED setting according to the WHO definitions. In this study, we demonstrated that standardization of mainly invasive procedures by the implementation of SOPs and introduction of flowcharts in combination with individual trainings on the job improve compliance significantly mainly by decreasing the number of HR needed. Finally, optimizing workflow practices seems to be a promising way to improve HH compliance without increasing workload, and thus represents an efficient solution to improve the quality of patient care and outcome. Since the study design was a before after trial, the results should be verified using an experimental study design.

## Competing interests

The authors declare that they have no competing interests.

## Authors’ contributions

SSC, PP and SL conceived the study and designed the trial. SL and RSR supervised the conduct of the trail. VK performed the observations during the observation phases. LLG performed the observations during the intervention phases. SSC supervised the data collection and managed the data including quality control. JB and CM managed the detailed performance including participating health-care workers and patients. RSR provided statistical advice on study design. SSC drafted the manuscript and all authors contributed substantially to its revision. SSC takes responsibility for the paper as a whole. All authors read and approved the final manuscript.

## Pre-publication history

The pre-publication history for this paper can be accessed here:

http://www.biomedcentral.com/1471-2334/13/367/prepub

## Supplementary Material

Additional file 1 Flowchart for the “patients‘way” in the ED. Click here for file
