# Supplementary figures and images for: Improving hand hygiene compliance in the emergency department: getting to the point
Source: BMC Infect Dis. 2013 Aug 7;13:367. doi: 10.1186/1471-2334-13-367 (PMC3750281; doi:10.1186/1471-2334-13-367)

# Appendix: Flowchart for the „patients`way“ in the ED

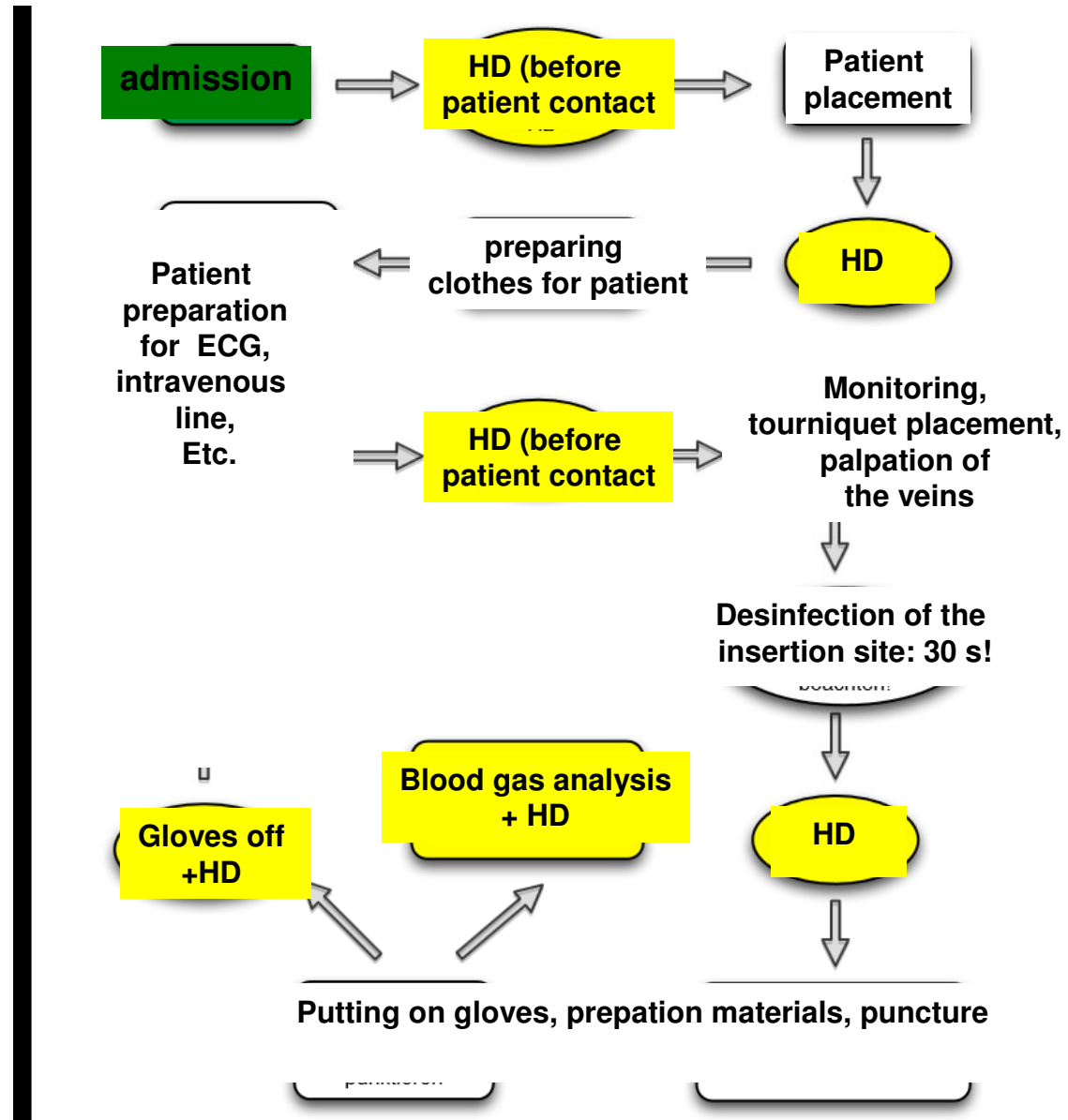

Supplement: Additional file 1 — Flowchart for the “patients‘way” in the ED. [file 1471-2334-13-367-S1.pdf]
